# Exploiting the Nutraceutical Potential of Polyphenol-Rich Extra Virgin Olive Oils Through Optimized Sustainable Phenolic Recovery

**DOI:** 10.3390/ijms27177925

**Published:** 2026-09-05

**Authors:** Athanasios Gerasopoulos, Diamanto Lazari

**Affiliations:** Laboratory of Pharmacognosy, Faculty of Health Sciences, School of Pharmacy, Aristotle University of Thessaloniki, 54124 Thessaloniki, Greece; ageraso@pharm.auth.gr

**Keywords:** phenolic compounds, extraction solvents, oleocanthal, oleacein, response surface methodology, DPPH, antioxidant

## Abstract

Recently developed polyphenol-rich extra virgin olive oils (EVOOs) are well-known for their health-promoting properties and provide the opportunity to extract polyphenolic content for innovative nutraceutical development. The extraction of phenolics from three polyphenol-rich EVOOs was optimized using response surface methodology (RSM) and ethanol/water mixtures. The effects of water content in ethanol and the solvent-to-oil ratio were evaluated based on results obtained using the Folin–Ciocalteu method. The extracts produced under the optimum conditions were also subjected to HPLC-DAD analysis and their phenolic profiles were obtained. The models derived showed high statistical significance (*p* ≤ 0.001) and a good fit to the experimental TPC data, as reflected by their R^2^ values of 94.41%, 96.25%, and 93.85%, for EVOOs -A, -B, and -C, respectively. Optimum extraction conditions (100% ethanol, ratio of 7.4–10:1) maximized phenolic yield of 1365.80, 1045.56, and 1005.67 mg Tyr. Eq./kg, for EVOOs -A, -B, and -C, respectively. The optimized extracts were validated in terms of their phenolic profiles, revealing high amounts of hydroxytyrosol, tyrosol, oleocanthal, oleuropein, ligstroside aglycone, and oleacein. These findings contribute to the efficient recovery of polyphenols from polyphenol-rich EVOOs for potential use in the development of innovative nutraceutical supplements, medicinal formulations, and/or functional foods.

## 1. Introduction

Recently, the use of plant matrices has expanded beyond direct consumption, with increased attention on processed products as sources of bioactive compounds that exhibit significant pharmacological and biological properties. Among these compounds, olive oil polyphenols have an established EU health claim (Regulation (EU) No. 432/2012) [1], which states that a daily consumption of 20 g olive oil (OL) containing more than 5 mg of OL polyphenols (OLPs) (hydroxytyrosol and its derivatives) contributes to the protection of blood lipids from oxidative stress.

OLPs account for about 2% of the total weight of OL [2] and are classified mostly as secoiridoids (demethyloleuropein, oleuropein, and ligstroside in their glycoside and aglycone forms). This fraction contains approximately 90% of the total phenolics. OL also contains phenolic alcohols (hydroxytyrosol, tyrosol, oleacein, and oleocanthal), flavonoids (luteolin and apigenin), lignans (i.e., (+)-pinoresinol), phenolic acids (i.e., p-coumaric, ferulic, cinnamic caffeic, gallic, vanillic, and syringic acid), and hydroxy-isocromans [3,4,5,6]. Moreover, OLP content can vary significantly among OLs, depending on a variety of factors such as fruit ripeness, geographical and genetic origin, irrigation, agronomic and pedo-climatic conditions, malaxation and extraction processes, as well as storage of the olive fruit and the obtained OL [7,8]. Reports on the phenolic content of OLs from the early 2000s frequently include levels in the range of 30–500 mg/kg OL [9,10,11,12,13,14,15]. However, over the past decade, as new insights and technological advances are introduced research has reported OLs with higher phenolic content levels, ranging from 1000 mg/kg OL to ~4500 mg/kg OL [16,17,18,19,20,21,22]. It is worth noting that recent interest in polyphenol-rich oils has resulted in new definitions for such oils. Diamantakos et al. [18] were the first to propose the terms “high” and “exceptionally high” for olive oils with phenolic content > 500 mg/kg and >1200 mg/kg, respectively, stating that these olive oils “will be able to retain the health claim limit (250 mg/kg) for at least 12 months after bottling”. Further, these high-phenolic EVOOs contain significantly higher levels of secoiridoids, particularly oleocanthal and oleacein, compared to olive oil industry by-products [4,18].

These results highlight an emerging opportunity to extract OLPs from polyphenol-rich (>500 mg/kg or >1000 mg/kg OL) OLs and use these to create novel products with high added value for both the pharmaceutical/supplement and food industries. The initial production of such items would necessitate an economically feasible and “green” OLP extraction process. Extensive research has been performed to extract the OLP fraction from OLs using both traditional and unconventional solvents/methods. In terms of practical procedures, liquid-to-liquid (LLE) extraction is the most common, with the official International Olive Oil Council (IOOC) recommending 80% MeOH [23] or a 4:1 MeOH-to-water ratio (*v*/*v*). However, this approach has not been developed for quantitative OLP extraction, nor has methanol been classified as generally recognized as safe (GRAS). Moreover, most reported OLP extraction processes that have been developed are intended for purposes of phenolic analysis. In this context, extraction processes such as solid-phase extraction (SPE), ultrasound-assisted extraction (UAE), microwave-assisted extraction (MAE), supercritical fluid extraction (SFE), pressurized liquid extraction (PLE), and the use of alternative solvents such as ionic liquids and deep eutectic solvents (DESs) have all been implemented [7,17,24,25,26,27,28]. However, for the purpose of maximizing phenolic recovery (from high-phenolic EVOOs) for downstream valorization, the required simple, efficient, relatively cheap, food-grade extraction strategy has not yet been reported. Ethanol is classified as GRAS, ICH (International Council for Harmonization of Technical Requirements for Pharmaceuticals for Human Use) class-3 (low hazardous potential), reasonably inexpensive, and it has a low boiling point (78.37C, easily evaporated). It has both hydrogen-bond donor (α = 0.88) and acceptor properties (β = 0.80), allowing it to interact with and solubilize a wide range of bioactive compounds. Finally, ethanol has been successfully used in ethanol/water mixture for the extraction of phenolics from olive leaves, a matrix that contains very similar phenolics to OL [29,30,31,32,33,34,35].

In this context, the goal of this research is to analyze and optimize a sustainable green extraction technique that uses ethanol/water systems to extract phenolics from polyphenol-rich extra virgin olive oils. For this reason, three EVOOs containing OLP > 1000 mg/kg OL were utilized to optimize OLP extraction applying response surface methodology (RSM) and evaluating various ethanol/water combinations and solvent–OL ratios.

The total phenolic content (TPC) of the produced OL extracts was determined, and the extracts were characterized for their primary phenolics using HPLC-DAD analysis and radical scavenging activity (DPPH). This study seeks to provide significant insights into the process for producing high-value-added products from high-phenolic EVOOs for the pharmaceutical and food industries.

## 2. Results and Discussion

### 2.1. Fitting the RSM Models

Extraction conditions were optimized through 14 randomized experimental runs to evaluate the effect of selected variables on the TPC yield of the three high-phenolic EVOOs. The experimental design (full central composite design—CCD) including the two independent variables and their corresponding coded and uncoded levels is presented in Table 1. The extraction procedure was optimized using a second-order polynomial equation. The models were highly significant (*p* < 0.001) and well fitted to the experimental data of TPC response, with R^2^ values of 94.41, 96.25, and 93.85% for EVOO-A, EVOO-B, and EVOO-C (Table 2). It should be highlighted that for all models, R^2^-adjusted and R^2^-predicted were less than 0.2, in contrast to R^2^, suggesting consistent accuracy and a significant correlation between observed and predicted data. Furthermore, lack-of-fit values were found to be non-significant (*p* > 0.050) for all produced models, showing that the models’ full central composite design accurately specified the relationship between the responses and the predictors (Table 2).

Multiple linear regression analysis was used to derive the regression coefficients for the dependent variables (Table 2). ANOVA results are presented in Supplementary Tables S1–S3. For all EVOOs examined, a significant (*p* < 0.001) negative linear effect of % water content (X_1_) was noted for TPC, while the opposite effect (*p* ≤ 0.001) was observed for the oil-to-solvent ratio (X_2_) parameter. The significance of the quadratic and interaction terms, however, varied among the three EVOOs. The quadratic effect of the ratio (X_2_^2^) was significant for EVOO-A (*p* = 0.007) and EVOO-B (*p* = 0.036) but not for EVOO-C (*p* = 0.703), whereas the quadratic effect of water content (X_1_^2^) was significant only for EVOO-B (*p* = 0.034) and non-significant for EVOO-A (*p* = 0.090) and EVOO-C (*p* = 0.244). The interaction term (X_1_X_2_) exhibited a significant negative effect only for EVOO-C (*p* = 0.010), while it was non-significant for EVOO-A (*p* = 0.217) and EVOO-B (*p* = 0.213). Among the linear terms, water content (X_1_) exhibited the highest sum of squares for EVOO-A and EVOO-C, whereas for EVOO-B, the solvent-to-oil ratio (X_2_) contributed the largest sum of squares; in all cases, the two linear terms were the principal contributors to the TPC response.

**Figure 1:**
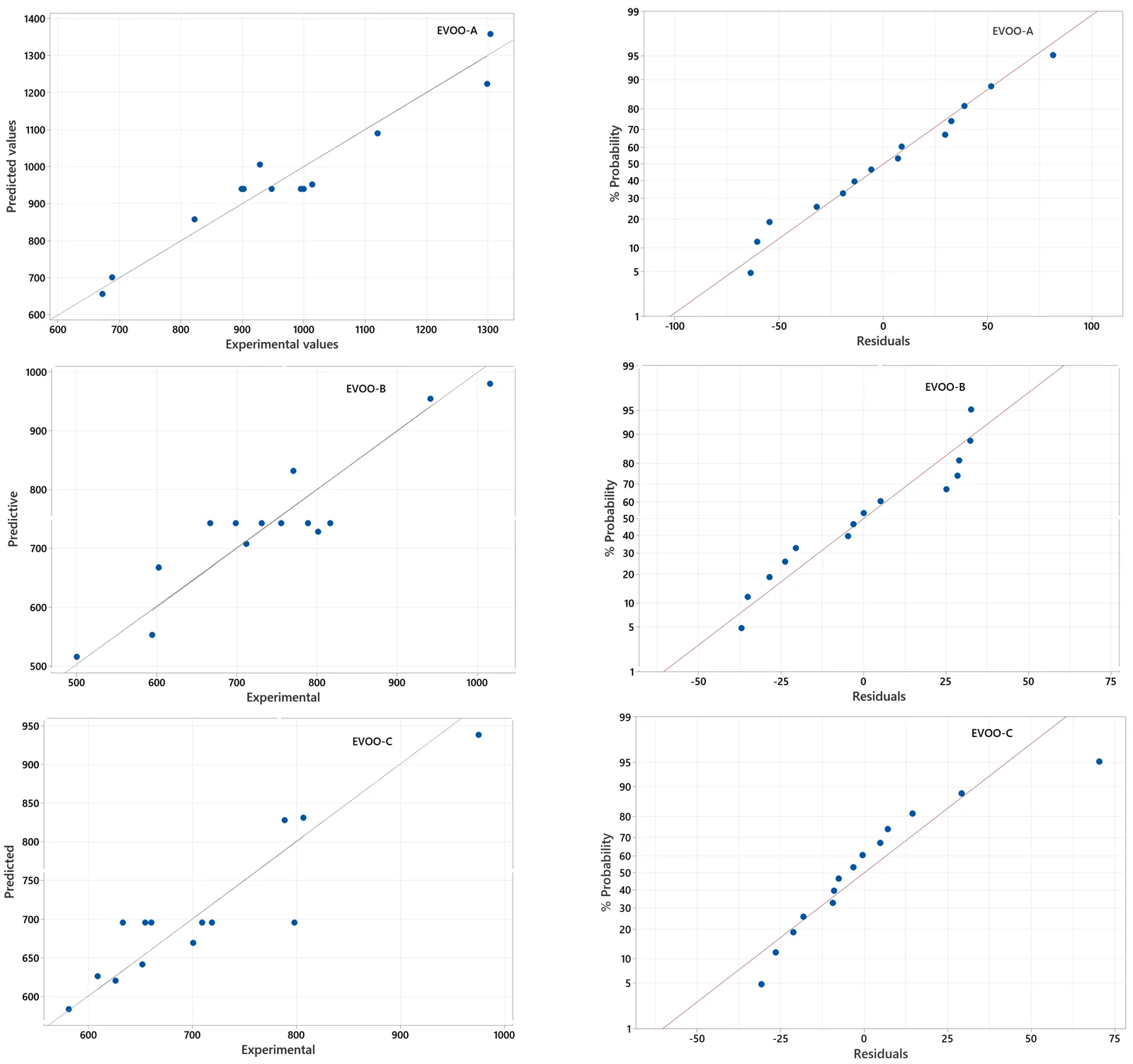
Actual vs. predicted values and normal plot of probability plots of the residuals for TPC measurementsof all EVOOs tested. In the actual vs. predicted plots, points represent the individual experimental runs and the line is the fitted regression; in the normal probability plots, points are the model residuals and the line is the theoretical normal distribution.

The adequacy of the models was further assessed by plotting the predicted and experimental TPC values (Figure 1). Finally, Table 3 displays both the models’ experimental and predicted values. For all EVOO samples, the experimental and predicted responses correlated well. Furthermore, the normal probability plots of the residuals revealed linear patterns, validating the normal distribution of errors and the model assumptions.

More specifically, the adequacy of the derived models was further evaluated through a complete residual diagnostics analysis, as shown in Supplementary Figure S1. Regarding the normal probability plots, it was observed that the residuals fell close to the reference line, in favor of the hypothesis of the normally distributed errors. This was further confirmed by the non-significant Anderson–Darling test results (*p* = 0.903, 0.148, and 0.097 for EVOO-A, -B, and -C, respectively). Moreover, for all EVOOs tested, the plots of residuals versus fitted values presented a random scatter around zero with no systematic pattern, indicating a homogeneity of the variance. It should be noted that the plots of the residuals versus observation order exhibited no clear trend, in support of the effectiveness of run randomization and independence of the residuals. Lastly, the residual histograms were approximately symmetric; taking into account the relatively limited number of runs (*n* = 14), they were interpreted qualitatively along with the normal probability plots.

### 2.2. Effect of Ethanol-to-Oil Ratio on Total Phenolic Content

The solvent–OL ratio is regarded as an important extraction characteristic because, on the one hand, it determines the quantity of bioactive chemicals partitioned from the oil matrix into the solvent. However, for handling, economic, and green chemistry objectives, the ratio should be as low as possible. In this investigation, the ratio parameter was found to have a significant impact on phenolic yield across all EVOOs examined, as evidenced by the ANOVA parameters presented in Supplementary Tables S1–S3.

**Figure 2:**
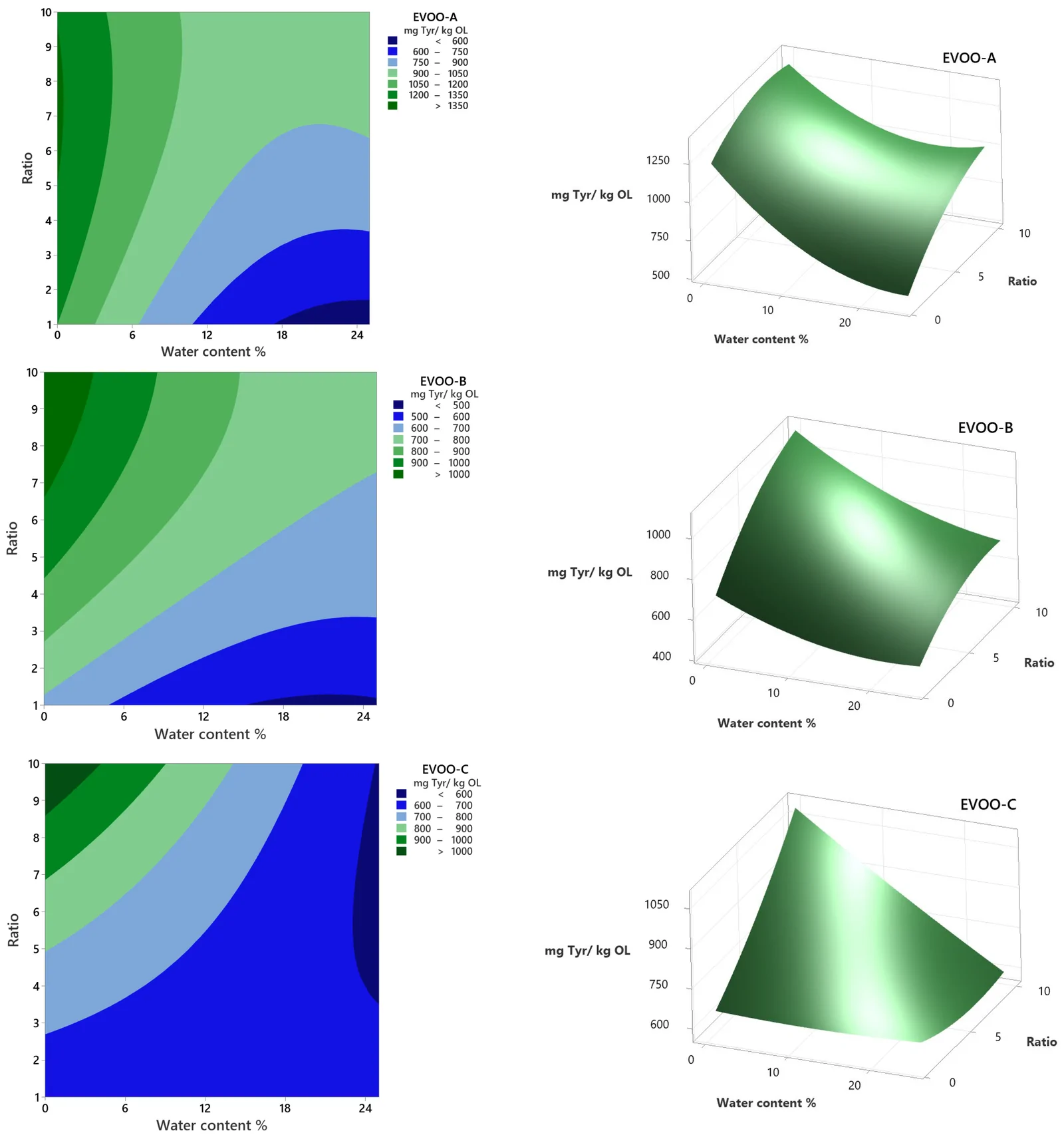
Response surface and contour plots (first extraction) showing the interactive effect of ethanol concentration and solvent-to-solid ratio on total polyphenol content (TPC) for EVOO-A, EVOO-B, and EVOO-C.

Contour and 3D-surface graphs (Figure 2) show the solvent-to-oil ratio in respect to the water content of ethanol. It was observed that increasing the value of ratio resulted in higher phenolic yields. It should be noted that in order to achieve the highest phenolic yield, ratio values were applied at the upper limit (Table 1). This type of interaction is prevalent among methodologies for extraction. An enhanced ratio normally permits the extractant to strongly interact and solubilize the analytes by efficiently entering the targeted plant matrix, implementing a larger concentration gradient [36,37,38]. Furthermore, it reduces the concentration of the analytes in the extractant phase, thereby decreasing their tendency to repartition into the EVOO and favoring their retention in the extractant at equilibrium. In the present system, this effect is also supported by the physicochemical properties of ethanol, as previously mentioned, and the nature of the OLPs that containin polar functional groups capable of interacting with ethanol (i.e. via hydrogen bonds). Although ethanol is partially miscible with olive oil, the high solvent–OL ratio increases the relative volume of the bulk ethanol phase. However, in certain cases, using an overwhelming ratio could lead to decreased yield, possibly due to oversaturation of extraction media, restricting mass transfer rates [39,40,41,42]. It should be noted that miscibility may also be influenced by the phospholipid content of the EVOO, which could consequently affect the phenolic yield [43]. However, phospholipids are minor constituents of olive oil, present at concentrations approximately 300–400 times lower than those reported for other seed oils [44]. In particular, phospholipid content in olive oil ranges from 11–157 mg/kg, with cloudy (veiled) oils exhibiting significantly higher phospholipid levels than naturally filtered oils, such as those used in the present study [45,46]. Moreover, the proposed optimized extraction procedure yielded near-complete quantitative recovery of phenolics across all EVOOs tested, further supporting the limited potential influence of phospholipids on the obtained results.

Moreover, the possibility of emulsification emerges due to the addition of excessive solvent volumes, hindering physical phase separation. Nevertheless, all ethanol/water mixtures achieved phase separation after centrifugation across the tested ratios. Furthermore, an increased ratio would require greater energy to concentrate or evaporate the solvent, thereby imposing a practical limitation on downstream processing. Lastly, it should be noted that reported liquid–liquid extraction of phenolics from EVOOs may also include the use of deep eutectic solvents (DESs), generally of greater solvent capacity than conventional solvents, applying a solvent-to-oil ratio of 1:1 [26,47]. In particular, Fanali et al. [48] proposes a ratio of 1:1 as the most efficient when applying a DES consisting of betaine–glycerol (1:2 molar ratio) with 30% water. However, none of the reported studies have aimed at the quantitative extraction of phenolics. Rather, they provide data regarding their relative extraction efficiency, often compared to the standard IOC extraction procedure using 80% MeOH solvent at a 3:2 solvent–oil ratio.

Furthermore, the effect of ratio demonstrated a definite trend with minor variations among the evaluated EVOOs. For EVOO-A, a ratio of 1:6–1:9 was adequate to reach maximum yield, whereas for EVOO-B and EVOO-C, ratios of 1:7–1:10 and 1:9–1:10, respectively, produced similar results. This could be attributable to a decreased phenolic content and/or compositional variations, which could cause challenges in analyte solubilization within the oil matrix. Olive oil polyphenols include compounds with varied polarities, whereas oxidation and hydrolytic processes during production and storage lead to the breakdown of complex secoiridioids and a subsequent increase in simple phenolics that are more hydrophilic [49,50].

**Figure 3:**
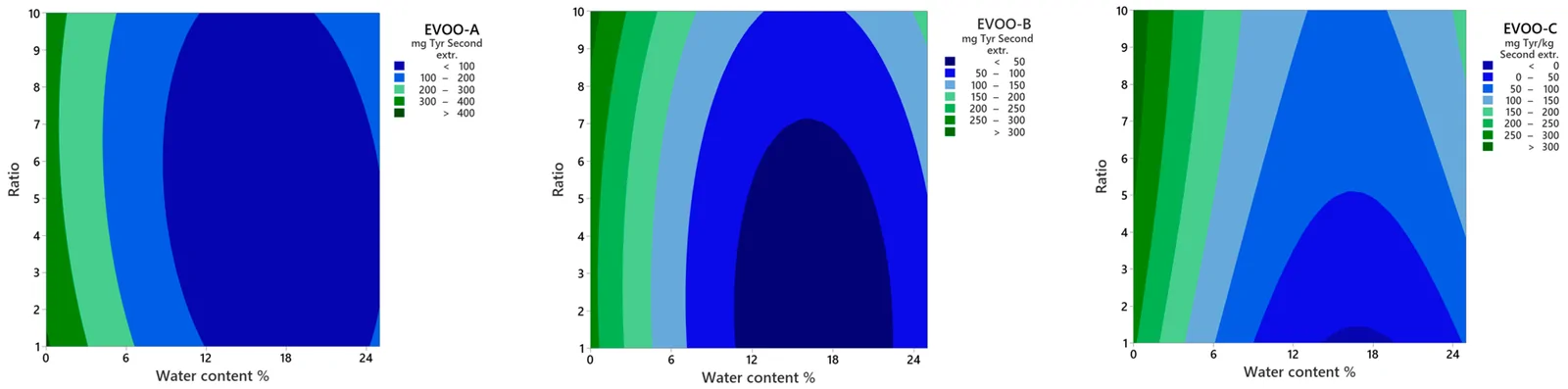
Contour plots for the second extraction of phenolics showing the interactive effect of ethanol concentration and solvent-to-solid ratio on total polyphenol content (TPC) for EVOO-A, EVOO-B, and EVOO-C.

The contour graph generated for the TPC data for the second extraction is shown in Figure 3. Maximum yields of 420.75 ± 22.92, 348.94 ± 6.11 and 390.19 ± 16.81 mg Tyr eq./kg for EVOO-A, EVOO-B, and EVOO-C, respectively, were obtained when extraction conditions including a ratio of 5.5:1 and pure ethanol were used (Supplementary Table S4). It was observed that, in the case of the second extraction, ratio values near the lower limit of the design may have recovered some of the residual OLPs in the EVOOs. However, the yield obtained from the first extraction (~80%) substantially exceeded that of the second extraction (~15–20%), highlighting a potential economic limitation associated with increased ethanol usage. According to the data presented in Supplementary Table S5, the third extraction yielded less than 1% of phenolics, while the fourth extraction using 100% methanol, despite its different polarity compared to ethanol/water mixtures, resulted in essentially zero phenolic yield. These findings indicate that two successive extractions are sufficient to achieve exhaustive phenolic recovery.

It is important to note that ethanol/water mixtures can be evaporated under vacuum at low temperatures (30–40 °C) without risking thermal degradation of phenolic compounds, allowing solvent recycling with minimal losses.

Additionally, the raw materials utilized in the preparation of EVOOs were from several cultivars; the effect of cultivar on the polyphenol profile of EVOO is of interest, as evidenced by the literature [51,52,53]. In particular, OL produced from Lianolia var. has been found to have generally greater quantities of the more lipophilic OLPs oleocanthal and oleacein than Koroneiki or Agrielia (wild) vars. [18,54]. Thus, a higher solvent-to-oil ratio may be required to successfully solubilize more lipophilic molecules.

### 2.3. Effect of Water Content on Total Phenolic Content

The influence of water content in the prepared ethanol/water mixtures on extract TPC was found to be statistically significant, as shown in the ANOVA tables (Supplementary Tables S1–S3). The produced surface and contour plots in Figure 1 show that as the water content diminished, the TPC of the extracts increased for all EVOOs tested. Pure ethanol with no water content produced peak TPC values of 1304 ± 33.61, 1016.27 ± 60.37, and 975.30 ± 48.15 for EVOO-A, EVOO-B, and EVOO-C, respectively. Adding less than ~%6 water to EVOO-B and EVOO-C extracts did not affect their TPC in the desired ratios. In contrast, EVOO-A with insignificant water content (<1%) had a lower TPC. The same pattern regarding % water content in ethanol was observed for the second extraction, where pure ethanol yielded increased phenolics, as shown in Figure 3 and Supplementary Table S4.

As previously stated, this could have been related to the varying phenolic profiles of the EVOOs, with OLPs exhibiting a variety of polarity and partition characteristics in the ethanol/water–OL system. For example, olive oil contains important phenolic compounds such as oleocanthal, oleacein, and oleuropein, all of which contribute to its health-promoting characteristics [55]. These chemicals are mostly present in high-phenolic EVOOs and have a large molecular size and a lower polarity than hydroxytyrosol and tyrosol, as well as other phenolic compounds like ferulic acid and cinnamic acid, which are more polar and hydrophilic. This is supported by the determined partition coefficients for OLPs. More particularly, oleocanthal and oleacein had lower partition coefficients than in a hydrophilic phase, indicating higher lipophilicity (or oil solubility). Other phenolics, such as hydroxytyrosol and tyrosol, which provide considerable health benefits, are more soluble in water than in oil. Increasing the water fraction in the ethanol/water mixture could also alter the solvents’ polarity, interfacial tension, and emulsion formation, directly impacting the extraction efficiency. Although physical phase separation is reported in this study, in the context of the different experiments conducted, the possibility of micro-emulsion formation and phenolic entrapment due to increased water content may arise. It should be noted that increasing the water content decreases the partial miscibility of ethanol with the EVOO phase and may contribute to a reduction in the efficiency of phenolic transfer across the oil–extractant interface. In the case of olive leaves, which share common phenolics with olive oil, 60% EtOH was the best performing system for extracting phenolics [29], but similar studies for scale-up processes employed an ethanol/water mixture in their liquid–liquid extraction method [56]. Water extracted more hydroxytyrosol and phenolic acids from olive leaves than 50% ethanol, but less oleuropein and flavonoids due to their lower polarity [57]. Ethanol/water mixtures have been extensively used in the literature for the extraction of phenolic compounds from a range of plant matrices such as olive leaves and pomace, mango peels, lychee and longan seeds, black currant, propolis and cherry seeds, optimized in most studies for 50–70% ethanol [58,59,60,61,62,63]. However, as mentioned, the absence of water in the extraction system appears to enhance the phenolic yield. Tasioula-Margari and Tsabolatidou [64] compared the efficiency of pure methanol and 80% methanol/water for the extraction of phenolics from EVOOs, yielding similar results. This phenomenon has been reported elsewhere in the literature; optimized phenolic extraction from EVOO using ultrasound-assisted liquid–liquid extraction and methanol/water mixtures indicated that phenolic yields decreased as the water content increased (Klen and Vodopivec) [65]. Similarly, Jerman et al., [66] compared the same methanol/water systems for the ultrasound-assisted solid–liquid extraction of olive fruit phenolic compounds, indicating that pure methanol presented enhanced yields, particularly for oleuropein and derivative compounds. Moreover, Capriotti et al. [67] reported that the most efficient extraction solvent for olive oil polyphenols is highly dependent on compound polarity. Although 70% methanol/water was the best performing overall for highly polar and medium-polar phenolics, pure methanol or acetonitrile was more suitable for the extraction of the less polar fraction such as ligstroside aglycone and derivatives.

### 2.4. Optimization of Total Phenolic Content

The optimal extraction conditions were obtained by maximizing the TPC response and are shown in Table 4. Pure ethanol was introduced, and ratios of 1:7.4 and 1:10 were applied to EVOO-A, EVOO-B, and EVOO-C. The strong agreement between the predicted optimum and the experimental results confirms the validity of the proposed model (Table 4).

The produced extracts were also evaluated in terms of their radical scavenging activity through the DPPH assay, yielding exceptionally high %RSA values of 92.6 ± 1.05, 90.0 ± 1.71, and 88.2 ± 0.37 for EVOOs -A, -B, and -C, respectively. It should be highlighted that %RSA values for olive oil typically range between 5% and 40%, although some studies have reported values as high as 98% [31,68,69,70,71,72]. The measured %RSA could be attributed to the high phenolic content of the EVOOs tested, as well as their phenolic composition, since compounds such as oleocanthal exhibit strong antioxidant activity [73]. The optimal extracts’ phenolic profiles were determined using HPLC-DAD analysis and supported the presence of elevated concentrations of oleocanthal across all EVOOs.

To further validate the efficiency of the proposed optimum conditions, additional olive oil samples (EVOO-D, -E, -F) were subjected to both IOC liquid–liquid and ethanol/water-based extraction. Regarding the TPC results of methanol/water extracts, measured through the FC assay, values of 390.50 ± 3.36, 294.44 ± 2.38, 345.82 ± 5.77 mg Tyr. Eq./kg EVOO were obtained for EVOO-D, -E, and -F, respectively. However, ethanol/water-based extraction yielded values of 432.20 ± 7.62, 435.97 ± 6.47, 495.90 ± 7.02 mg Tyr. Eq./kg EVOO, for EVOO-D, -E, and -F, respectively. The differential TPC pattern based on FC assay between methanol/water-ethanol/water was similar to the one found with EVOO-A, -B, and -C, thereby confirming the enhanced yield attained with the suggested optimized extraction method.

### 2.5. HPLC-DAD Analysis of Phenolic Profiles

**Figure 4:**
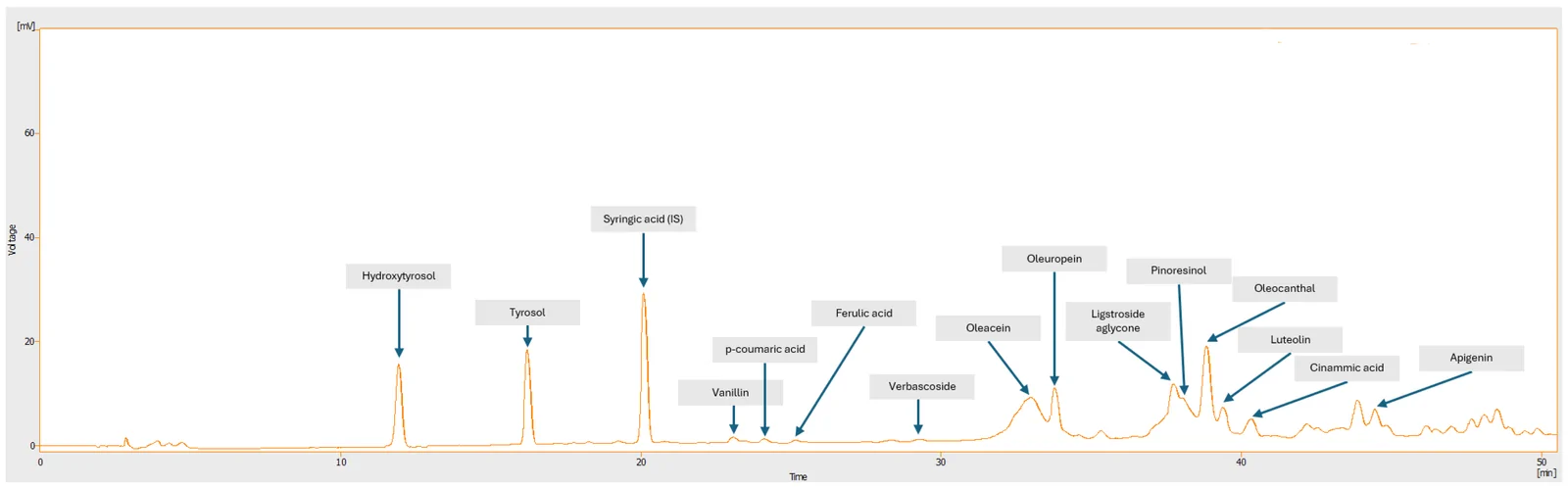
HPLC-DAD chromatogram of phenolic compounds extracted from EVOO-A under optimum conditions.

The phenolic profiles of EVOO-A, EVOO-B, and EVOO-C extracts (Figure 4 and Table 4) produced under optimum conditions (Table 5) or using the official IOC method [23] were determined using HPLC-DAD analysis. The total phenolics in EVOO extracts produced under the optimum conditions ranged from 1541.14 to 1195.43 mg/kg. Oleocanthal was the most abundant phenolic, accounting for 708.25 ± 11.29, 582.73 ± 3.27, and 606.38 ± 16.91 mg/kg for EVOO-A, EVOO-B, and EVOO-C, respectively. Also, oleacein concentration increased in EVOO-A, EVOO-B, and EVOO-C, reaching 294.47 ± 2.19, 260.24 ± 10.77, and 256.82 ± 0.35 mg/kg. These values fall within the concentration ranges reported in the literature for oleocanthal (not detected-711 mg/kg) and oleacein (not detected-500 mg/kg), respectively [4,68,74,75,76]. Furthermore, oleuropein content ranged from 77.17 to 105.37 mg/kg, in agreement with reported results ranging from not detected to 520 mg/kg [74,77]. Moreover, ligstroside aglycone exhibited high concentration levels, presenting a maximum recovery of 171.93 ± 0.99 mg/kg, similar to values reported in the literature for high-phenolic EVOOs [18,74].

It should be noted that the concentrations of the individual compounds quantified, as well as the comparative levels of oleocanthal and oleacein with hydroxytyrosol and tyrosol, are consistent with the time of harvest. Delayed harvesting yields lower phenolic content in the produced olive oil. Regarding the cultivar, EVOO-C (Lanolia var.) contained particularly high levels of oleocanthal [73,78,79]. Other minor substances with significant nutritional value include pinoresinol, a lignan, which was detected in concentrations ranging from 55.11 to 44.95 mg/kg, and luteolin, which was identified in concentrations ranging from 6.80–9.13 mg/kg. Values for pinoresinol (and generally lignans) vary widely in the literature (due to a number of factors, including the malaxation process), in the range of ND to 0.45–124.02 mg/kg [80,81,82,83,84,85,86,87]. For luteolin, values were within the reported range of 0.12–38.0 mg/kg found in olive oil [78,82,88,89,90].

Additionally, the HPLC-DAD phenolic profiles of the examined EVOO-D, -E, and -F revealed a similarities with EVOO-A, -B, and -C (Supplementary Table S6). However, due to prolonged storage and the expected degradation of phenolics over time, decreased levels of oleocanthal and oleacein, in particular, as well as comparatively increased concentrations of hydroxytyrosol and tyrosol, were evident [48,50].

Furthermore, Table 5 shows that the improved extraction devised in this study yielded more phenolics than the approved IOC method (which uses 80% MeOH) [23]. It should be noted that the IOC method was developed for analytical determination rather than quantitative recovery. However, it is widely used as a reference method in the literature and has been validated for its ability to efficiently extract the major phenolic compounds present in EVOO [9]. There are also significant differences in TPC evaluation between the colorimetric Folin–Ciocalteu method and the HPLC-DAD method, with the latter revealing values up to twice as high. This could be attributable to the HPLC-DAD method’s higher selectivity compared to the Folin–Ciocalteu method, as well as the fact that the FC method expresses its results relative to a reference compound, and differences in the specific molar response of phenolics may also contribute to reported variability in TPC values [91]. Olmo-García et al. [92] and Ricciutelli et al. [93] found similar conclusions after thoroughly examining the identified differences. 

In fuller detail, reported comparisons between the FC method and chromatographic methods, such as HPLC-DAD and LC-MS/MS, for the determination of TPC in olive oil and by-products have, although revealing a correlation of the methods, yielded contradictory results in terms of absolute values. While some studies noted higher TPC values according to the FC method than with chromatographic techniques, others observed the opposite or found no significant differences between the methods [94,95,96,97,98,99,100,101,102]. These inconsistencies mainly derive from the different analytical principles of quantification and limitations of the methods employed.

To elaborate on the FC method, the reagent utilized, phosphomolybdic–phosphotungstic acid, is reduced during a derivatization process, depending on its capacity to oxidize phenolic and non-phenolic organic and inorganic substances, such as ascorbic acid, amines, and sugars, present on the sample matrix [91]. This could lead to over- or underestimation of TPC. In contrast, the HPLC-DAD method relies on the availability of standards. However, due to the lack of standards for the different oleuropein and ligstroside derivatives or general secoiridoids of more complex forms [95] found in olive oil, these are not quantified and therefore, the HPLC-DAD method could underestimate the TPC. Alternatively, HPLC-DAD could yield more TPC than the FC method. Nevertheless, Alessandri et al. [96] reported a strong correlation between FC and HPLC-DAD measurements of TPC, despite differences in their absolute values, suggesting that both methods provide consistent estimations regardless of the relative phenolic composition of the oil. It should be highlighted that the authors also demonstrated that the FC reagent exhibited different responses toward individual phenolics, reacting less efficiently with lignans and flavonoids, while showing a stronger response toward stable ortho-diphenols.

Moreover, similarly to our study, Antonini et al. [97] isolated pure EVOO phenolic standards to construct compound-specific calibration curves, allowing the quantification of major secoiridoids derivatives. Under these conditions, HPLC-DAD determination of TPC exceeded the value obtained by the FC assay, mainly because secoiridoids, including oleocanthal, oleacein, oleuropein aglycone derivatives, and lignans, constituted the predominant phenolic fraction. Conversely, Reboredo-Rodríguez et al. [98] reported no significant differences between total phenolic contents determined by acid hydrolysis-HPLC and the FC assay, irrespective of whether a hydrolysis step was applied. 

Considering the nutraceutical potential of OLPs, secoiridoids such as oleocanthal and oleacein have been reported to reduce oxidative LDL damage and improve cardiovascular risk markers [103]. Moreover, OLPs exhibit biological activities comparable to those of mild nonsteroidal anti-inflammatory drugs (NSAIDs) [104], mainly attributed to their ability to modulate inflammatory pathways, including the inhibition of cyclooxygenase isoforms COX-1 and COX-2. In addition, secoiridoids have been investigated for their potential anticancer effects, including the inhibition of cell proliferation, induction of cell death (apoptosis, autophagy, and necrosis), suppression of angiogenesis and tumor metastasis, and modulation of cancer-associated signaling pathways [105]. Their recent evaluation in clinical studies has included interventions related to metabolic syndrome parameters in the OleoMetS study [106], early-stage chronic lymphocytic leukemia [107], mild cognitive impairment in the MICOIL pilot study [108], and oxidative status and inflammatory biomarkers in the OLIVAUS study [109]. This context implies the need for reliable quantification of individual bioactive compounds, which is essential for their commercialization as supplements, medicinal formulations, or food additives. Therefore, analytical limitations, such as the possible underestimation of total phenolic content by the Folin–Ciocalteu method, insufficient chromatographic resolution by HPLC-DAD, possible artifact formation due to chromatography mobile phase reactivity, and variability among quantification approaches, should be carefully considered and clearly reported.

## 3. Materials and Methods

### 3.1. Chemicals

All reagents were of analytical grade. Ethanol (absolute, ≥99.8%, HPLC-grade) was purchased from Fisher Scientific (Leicestershire, UK). The HPLC mobile phase was prepared using water, methanol, and HPLC-grade acetonitrile from Chem-Lab (Zedelgen, Belgium). Folin–Ciocalteu phenol reagent, sodium carbonate, glacial acetic acid, syringic acid, DPPH (2,2-diphenyl-picrylhydrazyl), and HPLC standards (hydroxytyrosol, tyrosol, vanillin, p-coumaric acid, ferulic acid, oleuropein, oleacin, oleocanthal, cinnamic acid, verbascoside, luteolin, apigenin, syringic acid) were all purchased from Sigma-Aldrich, (St. Louis, MO, USA). A Kern 770 balance (Balingen, Germany) was used to prepare all liquid solutions gravimetrically, with a precision of ±0.0001 g.

### 3.2. Olive Oil Samples

This study used three Greek EVOOs for the optimization of extraction process: EVOO-A (Koroneiki var.), EVOO-B (Agrielia “wild” var.), and EVOO-C (Lianolia var.). These EVOOs were collected during the 2024–2025 harvest seasons in Kalamata, Zakynthos, and Corfu. All samples had achieved clarity naturally (through gravity) and they satisfied the European Regulation’s standards for EVOOs. All were advertised as “polyphenol-rich,” with a health claim under Regulation (EU) No 432/2012 [1]. EVOO-D, EVOO-E, and EVOO-F were collected during the 2025–2026 harvest season, analyzed in summer 2026, and consisted predominantly of the Chalkidiki var., the Koroneiki var., and a blend of cultivars, respectively. All EVOOs were analyzed for their phenolic content using both the IOC method [23] and the Folin–Ciocalteu method [106,107].

### 3.3. Green Extraction

After collection, all EVOOs were placed in 250 mL containers (amber glass bottles with Teflon-lined caps), filled to the top to avoid oxidation by the presence of air, and stored in a freezer (−18 °C) until use. Prior to use, the samples were defrosted overnight in the dark. The were homogenized using a magnetic stirrer for 15 min before extraction with ethanol/water solutions in varying ratios, according to the experimental design (Table 1). The mixtures were prepared by vortexing for 30 s. The extraction was conducted out using the approved IOC procedure [23], with minor adjustments. Firstly, 3 g of EVOO spiked with syringic acid was mixed with the appropriate solvents (ethanol/water) at the ratio indicated by the experimental design, and both mixtures were vortexed for 5 min, followed by sonication for 30 min (at 25 °C) at maximum power using a Bandelin Sonorex Digiplus water bath (Berlin, Germany). They were then centrifuged at 9000 g for 15 min (Hettich Universal, Tuttlingen, Germany) to separate the ethanol/water phases from the OLs. These were then utilized to determine the total phenolic compound. They were also used to determine the phenolic profile using HPLC-DAD. This process was carried out in triplicate for EVOO samples and three replications for each sample (*n* = 9). Three more extractions were performed for each sample in order to evaluate the remaining phenolics in EVOOs after the initial extraction; ethanol/water was utilized as a solvent for the second and third sequential extractions, while pure methanol was used for the fourth. 

### 3.4. Experimental Design

To investigate the impact of independent factors, a response surface approach was employed. Minitab Release 20 (Minitab, Inc., State College, PA, USA) software and a blocked full central composite design (CCD) were used to analyze the effects of water content (%) in ethanol (X_1_) and EVOO/solvent ratio (X_2_) on total phenolic content (Y_1_) at five experimental levels coded as −a, −1, 0, +1, +a (a = 1.41421). Five of the 14 experimental runs that made up CCD were carried out at the center point and duplicated for error estimate (Table 1). To optimize the impact of unexplained variability in observed responses resulting from unrelated causes, the experiments were randomized.

A second-order polynomial model was fitted to the data:

Y = β_0_ + β_1_X_1_ + β_2_X_2_ + β_11_X_1_^2^ + β_22_X_2_^2^ + β_12_X_1_X_2_ + ε (1)

where Y is the response, X_1_ and X_2_ are coded variables, and β are the regression coefficients.

ANOVA was used to assess the quality of fitted models, including R^2^ values, p-values, and lack of fit. Terms with *p*-values < 0.050 were considered significant. A simplified model was generated by systematically deleting non-significant factors while maintaining the model hierarchy. The adequacy of the models was further assessed through residual analysis.

### 3.5. Optimization of Extraction

Minitab software (Release 20-Minitab, Inc., State College, PA, USA) was also used to optimize multiple responses to find the optimal combination of criteria. The predicted optimal conditions were then verified through experimentation to confirm the model’s accuracy for total phenolic compounds using the Folin–Ciocalteu [110,111] method. These samples were also tested for DPPH radical scavenging capacity and HPLC-DAD phenolic profile analysis.

### 3.6. Analyses

#### 3.6.1. Determination of Total Phenolic Content

Folin–Ciocalteu reagent as reported by Singleton et al. [112] and Scalbert et al. [113], with some modifications, was used to colorimetrically determine the total phenol content. Ethanol/water mixtures were used as controls, and 100 μL was diluted with 2.9 mL deionized water, then mixed with 0.25 mL Folin–Ciocalteu reagent and allowed to reactfor 1 min, after which 0.75 mL of Na_2_CO_3_ (20% *w*/*v*) was added, and finally stored in a dark place at room temperature (at 20 °C) for 60 min. A Genesys 180 UV-VIS spectrophotometer (Thermo Fisher Scientific, Waltham, MA, USA) was used to measure the solution’s absorbance at 760 nm. The linear regression equation of the standard curve (y = 0.0301x + 0.0261, R^2^ = 0.990) was used to calculate total phenolic compounds (TPCs), which were then expressed as tyrosol equivalents (Tyr eq. mg/kg). When required, the samples were diluted using an appropriate ethanol/water mixture; all EVOO extract analyses were conducted in triplicate (*n* = 9); and the reagents were handled in the coldest, darkest feasible conditions.

#### 3.6.2. Determination of Radical Scavenging Activity

With minor adjustments, the antiradical properties of the samples were determined using DPPH as a free radical according to Brand–Williams et al. [99] and Nenadis and Tsimidou [100]. In a test tube, 3.9 mL of DPPH methanolic solution (100 μM) was mixed; 0.1 mL of ethanol alone was used as a control. After that, the tubes were vortexed and left at room temperature (at 20 °C) for 30 min in a dark environment. A Genesys 180 UV-VIS spectrophotometer (Thermo Fisher Scientific, Waltham, MA, USA) was used to measure the absorbance of the solution at 517 nm. The radical scavenging antioxidant activity of the olive oil extracts, expressed as % values (%RSA), were determined using the following formula (after correction with appropriate blanks):

(2) $$\%\;RSA=\frac{\left({ABS}_{517\;\left(t=0\right)\;}-\;{ABS}_{17\;\left(t\right)}\right)}{{ABS}_{517\;\left(t=0\right)\;}}\;\times 100$$

where ABSrefers to the absorbance of a blank sample (*t* = 0) and the absorbance of an analyzed sample (*t*). All measurements were carried out in duplicate (*n* = 9).

#### 3.6.3. HPLC-DAD Phenolic Profile Analysis

The MeOH/water and optimum ethanol/water extracts for each EVOO were analyzed using an HPLC-DAD system (ECOM, Prague, Czech Republic) with a Supelco Discovery HS C18 column (5 μ, 150 × 4.6 mm I.D., St. Louis, MO, USA) at 25 °C, according to the official method of the International Olive Oil Council for the quantification of phenolics in olive oil [23]. The elution was carried out in gradient mode using a three-phase organic solvent mixture composed of water acidified with 0.2% acetic acid (solvent A), methanol (solvent B), and acetonitrile. A linear gradient ran from 96% (A), 2% (B), and 2% (C) to 50% (A), 25% (B), and 25% (C) over 40 min; it was changed to 40% (A), 30% (B), and 30% (C) for 5 min; and then changed to 0% (A), 50% (B), and 50% (C) for 25 min, followed by 12 min re-equilibration to the initial solvent composition. The mobile phase flow rate was 1 mL/min, and each sample had an injection volume of 20 μL. All phenolic compounds were identified by comparing retention times to those of standards (hydroxytyrosol, tyrosol, vanillin, p-coumaric acid, ferulic acid, oleuropein, oleacein, oleocanthal, cinnamic acid, apigenin, luteolin, verbascoside, pinoresinol, and syringic acid). HPLC analyses of EVOO extracts were carried out in triplicate (*n* = 3).

The phenolic profile of the ethanol/water extract was determined using high-performance liquid chromatography (with syringic acid added to EVOO as an internal standard). After reconstitution with 80% aqueous MeOH (1 mL) and 5 mL hexane, the mixture was vortexed and centrifuged at 6000× *g* for 15 min (Hettich Universal, Tuttlingen, Germany). Following phase separation, the hexane phase was removed, and the aqueous MeOH phase was filtered through a 0.45 mm PVDF filter before being analyzed directly using HPLC-DAD.

## 4. Conclusions

Ethanol/water extraction techniques were successfully used and optimized to extract phenolic compounds from polyphenol-rich EVOOs as a matrix. A maximum solvent-to-oil (*v*:*v*) ratio of 10:1 was determined to be optimal for maximizing extraction yield; however, ethanol/water mixtures resulted in yield losses. In a single extraction, ethanol solubilized an appreciable percentage of 85% all of the phenolics found in the polyphenol-rich EVOOs tested. This was confirmed using HPLC-DAD profile analysis.

Using ethanol in the extraction process is considered not only green but also industry-friendly, because ethanol can be recovered and reused in the extraction system with low loss. It should be noted that using ethanol to extract phenolics from EVOOs may solubilize other beneficial components from olive oil, such as tocopherols and squalene, as well as free fatty acids, which decrease the olive oil’s quality after extraction. However, this element is not covered in this study. Furthermore, ethanol is partially miscible with olive oil, which may result in unintentional solvent losses in larger quantities. Despite this limitation, as mentioned earlier, the enhanced solvent–OL ratio and the near-complete recovery of phenolics from the examined EVOOs indicate that the extracted phenolics preferentially interacted with and partitioned into the bulk ethanol phase, where they remained dissolved, rather than being retained within the ethanol fraction dispersed in the EVOO phase. The presence of ethanol residues in the residual oil may affect the quality of EVOOs following ethanol extraction; ethanol is considered as a substrate for the chemical synthesis of fatty acid ethyl esters (FAEEs), a raw material for mild deodorization, and an indication of fraude.

A sustainable and environmentally friendly quantitative extraction procedure of olive oil polyphenols could result in the development of unique functional foods and/or dietary supplements targetting key diseases that polyphenols have been shown to prevent or treat.

## Figures and Tables

**Table 1:** Experimental design for two-factor-five-level CCD.

Factor	Variable	Levels				
		Coded values ^1^				
		−a	−1	0	1	+a
		Actual values				
Water content (%)	X_1_	1	2.31	5.5	8.68	10
Solvent–EVOO ratio	X_2_	0	3.66	12.5	21.33	25
		Run Order	X_1_	X_2_	Coded values	
		1	0	5.5	−1.414	0
		2	12.5	10	0	1.414
		3	12.5	5.5	0	0
		4	12.5	5.5	0	0
		5	25	5.5	1.414	0
		6	12.5	5.5	0	0
		7	12.5	1	0	−1.414
		8	12.5	5.5	0	0
		9	12.5	5.5	0	0
		10	3.66	2.31	−1	−1
		11	21.33	2.31	1	−1
		12	3.66	8.68	−1	1
		13	12.5	5.5	0	0
		14	21.33	8.68	1	1

^1^ Coded value = [actual level − (high level + low level)/2]/(high level − low level)/2.

**Table 2:** Quadratic regression models and statistical parameters for the response (total phenolic content—TPC).

Sample	2nd Order Polynomial Equation	Regression (*p*-Value)	R^2^	R^2^ (Adjusted)	R^2^ (Predicted)	Lack of Fit
EVOO-A	y = 940.4 − 176.6X_1_ + 107.5X_2_ + 84.0X_1_^2^ − 43.4X_2_^2^ + 40.7X_1_X_2_	0	94.41	89.61	73.04	0.302
EVOO-B	y = 742.9 − 101.4X_1_ + 111.8X_2_ + 34.0X_1_^2^ − 34.4X_2_^2^ − 24.4X_1_X_2_	0	96.25	93.03	74.72	0.31
EVOO-C	y = 695.8 − 86.5X_1_ + 72.5X_2_ + 5.2X_1_^2^ − 16.6X_2_^2^ − 62.1X_1_X_2_	0.001	93.85	88.57	74.34	0.504

**Table 3:** Experimental and predicted data for the total phenolic content (mg Tyr. Eq./kg EVOO).

Run	Water Content (%)-X_1_	Ratio (Solvent–OL)-X_2_	Experimental Values									Predicted Values		
			TPC (mg Tyr eq./kg EVOO)											
			EVOO-A			EVOO-B			EVOO-C			EVOO-A	EVOO-B	EVOO-C
1	0	5.5	1304.00	±	33.61	941.72	±	6.11	788.94	±	6.32	1358.20	954.25	828.38
2	12.5	10	928.89	±	55.56	770.56	±	75.00	806.66	±	52.24	1005.75	832.04	831.46
3	12.5	5.5	898.94	±	6.11	666.72	±	42.78	654.50	±	32.74	940.45	742.86	695.75
4	12.5	5.5	947.83	±	2.78	730.89	±	42.78	660.61	±	26.99	940.45	742.86	695.75
5	25	5.5	822.56	±	3.06	602.56	±	3.06	581.20	±	20.24	858.65	667.35	583.85
6	12.5	5.5	999.78	±	30.56	698.81	±	38.19	633.10	±	8.53	940.45	742.86	695.75
7	12.5	1	688.17	±	20.28	500.11	±	13.33	609.00	±	9.88	701.60	515.95	626.37
8	12.5	5.5	902.00	±	36.67	816.44	±	15.28	798.10	±	23.86	940.45	742.86	695.75
9	12.5	5.5	995.19	±	59.58	755.33	±	76.39	718.70	±	60.89	940.45	742.86	695.75
10	3.66	2.31	1120.00	±	20.60	711.76	±	6.30	700.80	±	39.67	1090.87	707.72	669.33
11	21.33	2.31	672.48	±	55.40	594.57	±	30.26	626.10	±	55.53	656.16	553.57	620.67
12	3.66	8.68	1298.44	±	64.27	1016.27	±	60.37	975.30	±	48.12	1224.47	979.95	938.60
13	12.5	5.5	898.94	±	33.61	788.94	±	67.89	709.50	±	68.98	940.45	742.86	695.75
14	21.33	8.68	1013.86	±	13.45	801.63	±	77.17	652.10	±	2.35	952.70	728.36	641.45

**Table 4:** Experimental data of the validation of predicted values for TPC response (mg Tyr. Eq./kg EVOO) at optimal extraction conditions and corresponding %RSA values.

Sample	Water Content (%)-X_1_	Ratio (Solvent–OL)-X_2_	Response	Predicted Value	Prediction Interval (95%)	Experimental Value			Confidence Interval (95%)	%CV	%RSA		
EVOO-A	0	7.4	TPC	1372.56	1181.35–1563.77	1365.80	±	15.56	1244.54–1500.58	0.49	92.6	±	2.05
EVOO-B	0	10		1092.16	942.57–1241.75	1045.56	±	10.38	968.42–1215.90	4.46	90.0	±	1.71
EVOO-C	0	10		1088.34	939.42–1237.25	1005.67	±	11.99	965.16–1211.51	8.22	88.2	±	2.37

**Table 5:** Average concentrations (mg/kg) of the phenolic compounds investigated in the EVOOs by applying optimum conditions and the IOC method, following HPLC-DAD profile analysis and total phenolic content (TPC, mg Tyr. Eq./kg EVOO) according to the Folin–Ciocalteu assay.

Sample	EVOO-A Optimum			EVOO-A IOC			EVOO-B Optimum			EVOO-B IOC			EVOO-C Optimum			EVOO-C IOC		
Hydroxytyrosol	81.69	±	0.17	21.77	±	1.07	53.52	±	0.04	23.19	±	0.06	44.73	±	2.76	18.07	±	1.10
Tyrosol	126.93	±	1.64	17.34	±	0.07	94.81	±	1.93	38.55	±	0.51	72.03	±	0.75	13.68	±	1.12
Vanillin	2.72	±	0.14	1.88	±	0.00	2.51	±	0.32	2.07	±	0.02	2.11	±	0.08	1.85	±	0.00
p-coumaric acid	3.90	±	0.01	2.19	±	0.04	2.27	±	0.16	2.09	±	0.03	2.77	±	0.72	2.10	±	0.01
Ferulic acid	1.95	±	0.00	1.94	±	0.02	1.79	±	0.01	1.82	±	0.02	ND	±	0.00	ND	±	0.00
Verbascoside	2.16	±	0.02	1.13	±	0.06	0.94	±	0.02	1.04	±	0.07	0.92	±	0.01	0.91	±	0.00
Oleacein	294.47	±	2.20	215.27	±	18.26	260.24	±	10.78	163.83	±	0.06	256.82	±	0.35	61.68	±	8.49
Oleuropein	105.40	±	0.38	83.57	±	4.38	81.50	±	0.10	60.72	±	0.77	77.18	±	2.59	20.40	±	4.22
Ligstroside aglycone	141.40	±	0.99	90.87	±	1.07	118.97	±	1.53	66.92	±	1.83	76.59	±	2.12	45.71	±	1.73
Pinoresinol	55.12	±	0.38	22.57	±	1.19	56.34	±	1.87	24.54	±	1.11	44.95	±	3.08	16.94	±	0.82
Oleocanthal	708.25	±	11.29	475.83	±	21.89	582.73	±	3.27	151.01	±	24.89	606.38	±	16.91	208.99	±	4.40
Luteolin	9.13	±	0.04	5.54	±	0.05	8.97	±	0.01	6.81	±	1.31	6.81	±	1.31	11.47	±	1.54
Cinnamic acid	8.02	±	0.01	7.69	±	0.61	2.89	±	0.05	0.25	±	0.05	4.15	±	1.73	2.02	±	0.75
Apigenin	4.96	±	0.02	1.97	±	0.05	3.12	±	0.11	1.18	±	0.03	3.01	±	0.07	1.06	±	0.07
Total (mg/kg)	1541.14			947.59			1267.50			542.82			1195.43			403.82		
TPC (mg Tyr. Eq./kg EVOO)	1365.80	±	15.56	699.11	±	8.11	1045.56	±	10.38	383.00	±	6.11	1005.67	±	11.99	344.11	±	12.78

## Data Availability

The original contributions presented in this study are included in the article/Supplementary Materials. Further inquiries can be directed to the corresponding author.

## Supplementary Materials

The following supporting information can be downloaded at https://www.mdpi.com/article/10.3390/ijms27177925/s1.
