# Supplementary material for: Exploiting the Nutraceutical Potential of Polyphenol-Rich Extra Virgin Olive Oils Through Optimized Sustainable Phenolic Recovery
Source: Int J Mol Sci. 2026 Sep 5;27(17):7925. doi: 10.3390/ijms27177925 (PMC13566635; doi:10.3390/ijms27177925)
Supplement: Supplementary file 1 [file ijms-27-07925-s001.zip › ijms-4431014-supplementary.pdf]

Exploiting the nutraceutical potential of Polyphenol-Rich Extra Virgin Olive Oils through Optimized Sustainable Phenolic Recovery

Athanasios Gerasopoulos, Diamanto Lazari*

Laboratory of Pharmacognosy, Faculty of Health Sciences, School of Pharmacy, Aristotle University of Thessaloniki, 54124 Thessaloniki, Greece; ageraso@pharm.auth.gr

* Correspondence: dlazari@pharm.auth.gr; Tel.: (optional; include country code; if there are multiple corresponding authors, add author initials)

Supplementary Material

Table S1. ANOVA table for EVOO-A.

Source	Sum of squares	df	Mean square	F value	P value
Model (EVOO-A)	426144	6	71024	19.69	< 0.001
Blocks	6898	1	6898	1.91	0.209
X_1	249557	1	249557	69.17	< 0.001
X_2	92503	1	92503	25.64	0.001
X_2^2	52091	1	52091	14.44	0.007
X_1^2	13901	1	13901	3.85	0.09
X_1X_2	6637	1	6637	1.84	0.217
Residual	25253	7	3608	-	-
Lack of fit	14182	3	4727	1.71	0.302
Pure error	11071	4	2768	-	-
Cor total	451397	13	-	-	-

Table S2. ANOVA table for EVOO-B.

Source	Sum of squares	df	Mean square	F value	P value
Model (EVOO-B)	226818	6	37803	29.91	< 0.001
Blocks	23501	1	23501.5	18.6	0.004
X_1	82312	1	82312.1	65.13	< 0.001
X_2	99914	1	99914.1	79.06	< 0.001
X_2^2	8523	1	8522.6	6.74	0.036
X_1^2	8754	1	8754	6.93	0.034
X_1X_2	2374	1	2374.1	1.88	0.213
Residual	8847	7	1263.8	-	-
Lack of fit	4915	3	1638.2	1.67	0.31
Pure error	3932	4	983.1	-	-
Cor total	235665	13	-	-	-

(a)

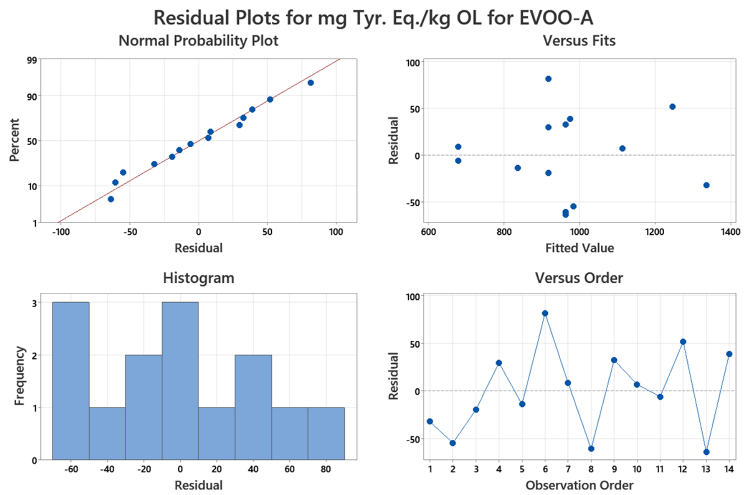
Residual Plots for mg Tyr. Eq./kg OL for EVOO-A
Normal Probability Plot
Percent
Residual
Versus Fits
Residual
Fitted Value
Histogram
Frequency
Residual
Versus Order
Residual
Observation Order

(b)

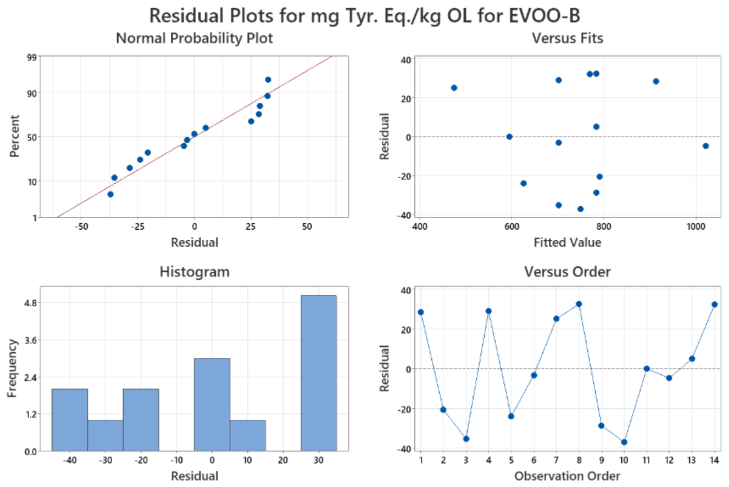
Residual Plots for mg Tyr. Eq./kg OL for EVOO-B
Normal Probability Plot
Percent
Residual
Versus Fits
Residual
Fitted Value
Histogram
Frequency
Residual
Versus Order
Residual
Observation Order

(c)

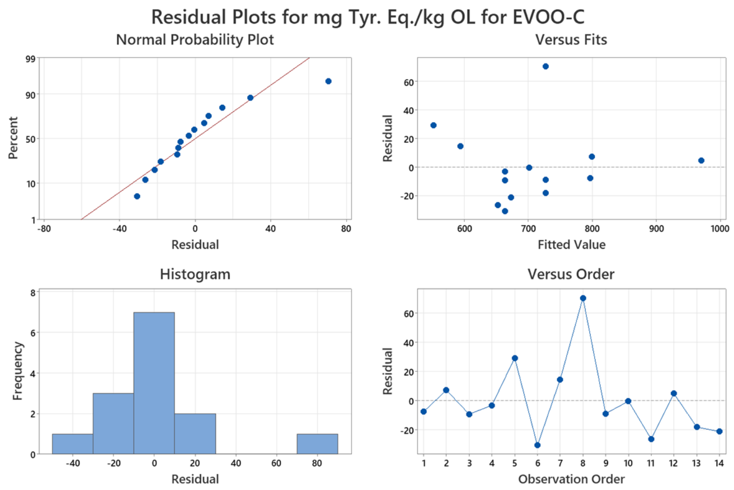

Figure S1. Residual diagnostic plots for the fitted second-order models of total phenolic content (TPC) for (a) EVOO-A, (b) EVOO-B and (c) EVOO-C. Normal probability plot of residuals, residuals versus fitted values, histogram of residuals, and residuals versus observation order are presented.

Table S3. ANOVA table for EVOO-C.

Source	Sum of squares	df	Mean square	F value	P value
Model (EVOO-C)	133686	6	22280.9	17.79	0.001
Blocks	14247	1	14246.5	11.38	0.012
X_1	59794	1	59794.4	47.74	< 0.001
X_2	42063	1	42063.1	33.59	0.001
X_2^2	198	1	198.2	0.16	0.703
X_1^2	2030	1	2030.3	1.62	0.244
X_1X_2	15438	1	15438.1	12.33	0.01
Residual	8767	7	1252.4	-	-
Lack of fit	3603	3	1201.1	0.93	0.504
Pure error	5163	4	1290.9	-	-
Cor total	142453	13	-	-	-

Table S4. Experimental data (TPC) for the second extraction.

Run	Water content (%)-X_1	Ratio (Solvent:matrix)-X_2	Experimental values (Second Extraction) TPC (mg CAE eq./kg EVOO)		
			EVOO-A	EVOO-B	EVOO-C
1	0	5.5	420.75 ± 22.92	348.94 ± 6.11	390.19 ± 16.81
2	12.5	10	26.11 ± 13.89	92.78 ± 6.35	67.78 ± 27.78
3	12.5	5.5	54.08 ± 1.53	47.97 ± 7.64	63.25 ± 16.81
4	12.5	5.5	41.86 ± 4.58	28.11 ± 2.00	80.06 ± 15.28
5	25	5.5	33.67 ± 0.39	54.08 ± 1.07	46.44 ± 6.11
6	12.5	5.5	142.69 ± 22.92	67.83 ± 1.99	41.86 ± 19.86
7	12.5	1	145.11 ± 12.22	69.56 ± 1.11	53.72 ± 3.06
8	12.5	5.5	110.61 ± 9.17	61.72 ± 2.67	78.53 ± 25.97
9	12.5	5.5	73.94 ± 6.11	32.69 ± 1.40	96.86 ± 1.53
10	3.66	2.31	158.01 ± 5.80	96.20 ± 5.80	83.32 ± 1.93

11	21.33	2.31	82.03	±	9.66	55.63	±	2.58	57.56	±	1.93
12	3.66	8.68	210.78	±	10.37	167.37	±	12.06	196.31	±	31.35
13	12.5	5.5	51.03	±	10.69	41.86	±	5.69	52.56	±	12.22
14	21.33	8.68	196.31	±	12.06	143.25	±	21.92	189.07	±	38.59

Table S5. Experimental data (TPC) for the third extraction.

Run	Water content (%)-X_1	Ratio (Solvent:matrix)-X_2	Experimental values (Third Extraction)								
			TPC (mg CAE eq./kg EVOO)								
			EVOO-A			EVOO-B			EVOO-C		
1	0	5.5	4.08	±	0.41	2.88	±	0.09	3.54	±	0.28
2	12.5	10	0.02	±	0.01	0.13	±	0.07	0.08	±	0.04
3	12.5	5.5	0.13	±	0.01	0.11	±	0.02	0.17	±	0.06
4	12.5	5.5	0.09	±	0.01	0.08	±	0.08	0.12	±	0.01
5	25	5.5	0.04	±	0.06	0.14	±	0.07	0.10	±	0.02
6	12.5	5.5	0.60	±	0.16	0.19	±	0.10	0.10	±	0.06
7	12.5	1	13.78	±	2.22	3.41	±	0.10	2.13	±	0.22
8	12.5	5.5	0.39	±	0.05	0.16	±	0.07	0.24	±	0.12
9	12.5	5.5	0.21	±	0.03	0.07	±	0.05	0.31	±	0.01
10	3.66	2.31	3.29	±	0.22	1.34	±	0.14	1.04	±	0.04
11	21.33	2.31	1.02	±	0.21	0.52	±	0.04	0.55	±	0.03
12	3.66	8.68	0.62	±	0.43	0.36	±	0.04	0.48	±	0.12
13	12.5	5.5	0.12	±	0.04	0.10	±	0.08	0.13	±	0.04
14	21.33	8.68	0.47	±	0.05	0.30	±	0.14	0.45	±	0.15

Table S6. Average concentrations (mg/kg) of the phenolic compounds investigated in the EVOOs (EVOO-D, -E and -F) by applying optimum conditions and the IOC method, following HPLC-DAD profile analysis and total phenolic content (TPC, mg Tyr. Eq./kg EVOO) according to the Folin-Ciocalteu assay.

Sample	EVOO-D Optimum			EVOO-D IOC			EVOO-E Optimum			EVOO-E IOC			EVOO-F Optimum			EVOO-F IOC		
Hydroxytyrosol	30.19	±	1.02	18.96	±	2.29	14.80	±	0.04	10.93	±	0.05	58.67	±	3.94	54.86	±	1.03
Tyrosol	69.97	±	1.14	48.64	±	0.07	37.32	±	1.93	32.12	±	0.57	190.34	±	1.12	172.26	±	1.02
Vanillin	1.85	±	0.14	1.05	±	0.02	4.96	±	0.32	4.00	±	0.02	6.20	±	0.02	5.16	±	0
p-coumaric acid	ND	±	0.00	ND	±	0.00	4.38	±	0.16	3.85	±	0.03	5.09	±	0.98	4.72	±	0.04
Ferulic acid	ND	±	0.00	ND	±	0.00	ND	±	0.00	ND	±	0.00	1.09	±	0.03	0.97	±	0
Verbascoside	ND	±	0.00	ND	±	0.00	0.27	±	0.02	0.19	±	0.08	ND	±	0.00	ND	±	0
Oleacein	129.88	±	2.52	108.51	±	9.87	102.42	±	10.78	62.35	±	0.05	77.70	±	1.66	24.53	±	7.18
Oleuropein	81.33	±	0.38	64.65	±	5.00	45.70	±	0.10	23.72	±	0.69	28.44	±	1.39	17.75	±	3.95
Ligstroside aglycone	118.47	±	0.99	70.00	±	3.24	120.47	±	1.53	90.31	±	2.02	46.79	±	2.12	16.84	±	1.48
Pinoresinol	10.53	±	0.38	4.95	±	0.32	7.44	±	1.87	4.58	±	0.99	44.95	±	4.09	21.68	±	0.74
Oleocanthal	271.78	±	11.29	141.94	±	17.30	173.48	±	3.27	92.6	±	27.31	124.251	±	6.17	47.98	±	3.64
Luteolin	4.94	±	0.04	3.19	±	0.04	6.16	±	0.01	3.01	±	1.13	ND	±	0.00	ND	±	1.43
Cinnamic acid	2.94	±	0.03	2.74	±	0.11	2.75	±	0.02	2.56	±	0.06	0.42	±	1.73	0.38	±	0.66
Apigenin	3.13	±	0.12	3.01	±	0.03	1.78	±	0.21	1.42	±	0.03	1.36	±	0.06	1.17	±	0.06

Total (mg/kg)	725.01			467.64			521.943			331.64			585.30			403.82		
TPC (mg Tyr. Eq./kg EVOO)	432.20	±	7.62	390.50	±	3.36	435.97	±	6.47	294.44	±	2.38	495.90	±	7.02	345.82	±	5.77
